# Intrarenal microRNA signature related to the fibrosis process in chronic kidney disease: identification and functional validation of key miRNAs

**DOI:** 10.1186/s12882-019-1512-x

**Published:** 2019-08-27

**Authors:** Jianwen Yu, Chaolun Yu, Boya Feng, Xiaojiang Zhan, Ning Luo, Xueqing Yu, Qin Zhou

**Affiliations:** 1 Department of Nephrology, The First Affiliated Hospital, Sun Yat-sen University, 58th, Zhongshan Road II, Guangzhou, China; 2 National Health Commission Key Laboratory of Nephrology, The First Affiliated Hospital, Sun Yat-sen University, Guangzhou, China; 3 Guangdong Provincial Key Laboratory of Nephrology, The First Affiliated Hospital, Sun Yat-sen University, Guangzhou, China; 4 Department of Endocrinology, Sun Yat-sen Memorial Hospital, Sun Yat-sen University, Guangzhou, China; 5 Translational Medical Center, The First Affiliated Hospital, Sun Yat-sen University, Guangzhou, China; 6 Department of Nephrology, The First Affiliated Hospital, Nanchang University, Nanchang, China; 7 Guangdong General Hospital, Guangzhou, China

**Keywords:** Intrarenal microRNAs, Chronic kidney disease, Renal fibrosis

## Abstract

**Background:**

Though the roles of microRNAs (miRNAs) in renal diseases have been extensively investigated, a thorough screening and comparison of miRNAs among different types of chronic kidney disease (CKD) has never been performed.

**Methods:**

The intrarenal miRNAs were profiled from fresh kidney tissues of patients with biopsy-proven minimal change disease (MCD), focal segmental glomerular sclerosis (FSGS) and diabetic nephropathy (DN) by using microarray. Commonly dysregulated miRNAs were validated by real-time PCR using paraffin-embedded renal tissues from all three types of CKD patients as well as mouse unilateral ureteral obstruction (UUO) model. Two novel miRNAs were selected and annotations of their target genes were performed using GO and KEGG pathway enrichment analysis. Biological functions of three two candidate miRNAs were explored in TGF-β1-induced cell model using human kidney proximal tubular cells (HK-2).

**Results:**

The kidney biopsy samples of three disease types represent different levels of damage and fibrosis, which were the mildest in MCD, moderate in FSGS, and the most severe in DN. 116 miRNAs were identified to be commonly dysregulated, including 40 up-regulated and 76 down-regulated in CKD tissues as compared with healthy donor kidney biopsy tissues. Two novel miRNAs, hsa-miR-3607-3p and hsa-miR-4709-3p, were verified as consistently differentially expressed among all three types of patient samples as well as in mouse model. In vitro, hsa-miR-3607-3p was repressed while hsa-miR-4709-3p was induced by TGF-β1 treatment. Inhibition of hsa-miR-3607-3p or overexpression of hsa-miR-4709-3p promoted TGF-β1-induced migration and F-actin assembling in HK-2 cells, which are characteristics of epithelial–mesenchymal transition (EMT). Further study identified that ITGB8 and CALM3 were the bona fide target genes of hsa-miR-3607-3p and hsa-miR-4709-3p respectively.

**Conclusions:**

The present identify a unique miRNAs profile that probably relates to the common fibrosis process of CKD. Results of our study suggest that hsa-miR-3607-3p and hsa-miR-4709-3p may represent as promising therapeutic targets against kidney fibrosis.

**Electronic supplementary material:**

The online version of this article (10.1186/s12882-019-1512-x) contains supplementary material, which is available to authorized users.

## Background

Chronic kidney disease (CKD) is a major public health problem worldwide as well as in China. The prevalence of CKD in China is approximately 10.8–19.1% [1–3], and the leading cause is primary glomerulonephritis [4], including IgA nephropathy (IgAN), minimal change disease (MCD), and focal segmental glomerular sclerosis (FSGS). On the other hand, secondary glomerular diseases, especially diabetic nephropathy (DN), have been increasing substantially in recent years [5]. Regardless of the underlying etiology, progression of CKD results in glomerular sclerosis and tubulointerstitial fibrosis termed renal fibrosis, the final common pathway leading to end stage renal disease (ESRD). The mechanisms that drive progression of renal fibrosis remain largely unclear. Transforming growth factor-β1 (TGF-β1) has been recognized as the master regulator in the pathogenesis of renal fibrosis [6]. However, strategies targeting at TGF-β1 signaling fail to yield satisfactory protective effects [7]. A thorough understanding of the mechanisms of renal fibrosis will contribute to the development of treatment strategies against kidney diseases.

MicroRNAs (miRNAs) are small endogenous non-coding RNAs that are involved in a variety of pathophysiological processes, including development, fibrosis, and tumor [8, 9]. They exert their biological functions mainly by post-transcriptionally regulating gene expression via base-pairing with the 3′ untranslated regions (3′ UTRs) of target mRNAs. Mounting evidence shows that miRNAs play an important role in renal diseases. Numerous intra-renal differentially-expressed (DE) miRNAs have been identified from kidney biopsy specimens of IgAN [10, 11], DN [12, 13], hypertensive nephrosclerosis (HTN) [14], and lupus nephritis (LN) [15]. We and others have demonstrated that many miRNAs are regulated by TGF-β1 signaling and act as downstream factors mediating anti- or pro-fibrotic effects [16–20]. Some of them are shown to participate in the process of epithelial–mesenchymal transition (EMT) [16, 19, 20], which is one of the most investigated and controversial topics in renal fibrosis. However, a thorough screening and comparison of CKD-related miRNAs by using biopsy samples from different types of kidney diseases has never been performed. Another important issue is that considerable discrepancy regarding expression profiles as well as roles of candidate miRNAs in renal diseases exists among previous studies [21, 22]. Therefore, the present study analyzed the miRNAs profiles in renal tissues from patients with biopsy-proven MCD, FSGS, and DN by using microarray analysis. The renal biopsy samples of three different pathological patterns represent different levels of renal damage and fibrosis, which were the mildest in MCD, moderate in FSGS, and the most severe in DN group. Moreover, common miRNAs which changed consistently among three types of kidney diseases were selected for further functional studies.

## Methods

### Subjects and sample collection

Fresh kidney biopsy samples were obtained from patients with biopsy-proven MCD, FSGS, and DN (*n* = 4 per group), for microarray profiling analysis. Four cases of fresh normal healthy kidney tissue from donor biopsy without apparent lesions served as control. Formalin-fixed paraffin-embedded (FFPE) kidney biopsy specimens of patients with MCD, FSGS, DN, and healthy donor kidney (*n* = 6 per group) were used for validation study. Patient inclusion and exclusion criteria were listed in Additional file 9: Table S1**.** Renal biopsy sections were blindly reviewed by an expert pathologist. The percentage of glomerular sclerosis and the score of tubulointerstitial fibrosis were quantified over the whole sections. Tubulointerstitial fibrosis was scored on 0–3 scales using the BANFF criteria adapted to the native kidney with a semiquantitative image analysis: 0, < 10% of cortical area; 1, 10 to 25% of cortical area; 2, 25 to 50% of cortical area; 3, > 50% of cortical area) [23]. Demographic and clinical data including age, gender, serum creatinine, and 24-h urine protein were collected at the time of kidney biopsy. Estimated glomerular filtration rate (eGFR) was calculated by the standard CKD-EPI equation. The study protocol was approved by the Institutional Review Board and Ethics Committee of The First Affiliated Hospital of Sun Yat-sen University. All subjects provided written informed consent.

### Mouse model of UUO

The mouse model of unilateral ureteral obstruction (UUO) was induced as previously described [24]. Briefly, healthy 8-week-old C57BL/6 J SPF male mice were purchased from the Laboratory Animal Center of Sun Yat-sen University (Guangzhou, China) and randomly divided into UUO model group and sham group with 6 mice in each group. Mice of the model group were subjected to left ureteral ligation, while another group of mice, sham operated on, served as control. Mice were killed and kidney tissues were harvested at day 7 after surgery for further analysis. All animal experiments were approved by the Committee on Animal Experimentation of Sun Yat-sen University and performed in compliance with the Guidelines for the Care and Use of Laboratory Animals of the university.

### RNA isolation and microarray profiling

Total RNA of fresh kidney biopsy tissues was isolated using Tri-Reagent (Invitrogen, Carlsbad, CA) and miRNeasy Mini Kit (Qiagen, Germany), according to the manufacturer’s protocol. Briefly, fresh kidney tissues were stored in Trizol reagent and total RNA was isolated using Tri-Reagent method. Then the miRNeasy Mini kit was adopted to purify microRNAs since the clear up step using columns. The total amount of 1 μg RNA for each sample was labeled using the miRCURY™ Hy3™/Hy5™ Power labeling kit and hybridized on the miRCURY™ LNA Array, which contains 3100 capture probes, covering all human, mouse and rat miRNAs annotated in miRBase 18.0. The samples were scanned by the Axon GenePix 4000B microarray scanner and images were then imported into GenePix Pro 6.0 software for grid alignment and data extraction. Replicated miRNAs were averaged and those with intensities ≥30 in all samples were chosen for calculating normalization factor. Expressed data were normalized by using the Median normalization. After normalization, significant differentially expressed (DE) miRNAs were identified through Volcano Plot filtering. Finally, hierarchical clustering was performed to show distinguishable miRNAs expression profiling among samples. For expression analysis, miRNAs were considered as differentially expressed if the fold change is > 1.5 and significant if the *p* value is < 0.05.

### Validation of candidate miRNAs by quantitative real-time PCR (qRT-PCR)

For validation of candidate miRNAs, total RNA was extracted from FFPE specimens of CKD and normal donor kidney biopsy by RecoverAll™ Total Nucleic Acid Isolation Kit for FFPE (Invitrogen) following the instructions. Total RNA was isolated from human FFPE sections and measured by Nanodrop-2000 (Thermo Fisher Scientific). The A260/A280 ratio was required to be 1.8–2.1. For reverse transcription, 10 ng of total RNA was used for each sample according to the protocol of TaqMan™ MicroRNA Reverse Transcription Kit (Applie Biosystems™, Foster City, CA). QRT-PCR was performed on ABI 7900 system by using TaqMan microRNA assay kit (Applie Biosystems™). The PCR program is 50 °C for 2 min, 95 °C for 10 min, 40 cycles of 95 °C 15 s and 60 °C 1 min. MiRNAs were extracted from mouse UUO kidney using the miRNeasy Mini kit. Fresh kidney cortex tissues were used. The miRNA quantification protocol of qRT-PCR is the same as above. Level of miRNAs was normalized to U6 snRNA in each sample.

### In situ hybridization (ISH) of target miRNAs

To detect the expression pattern and location of hsa-miR-4709-3p and has-miR-3607-3p in the kidney, in situ hybridization was performed in control and CKD kidneys using FFPE sections as described previously [24]. Specific LNA-digoxigenin labeled hsa-miR-4709-3p probe (5′-UUG AAGAGGAGGUGCUCUGUAGC-3′) and hsa-miR-3607-3p probe (5′-ACUGUAAACGCUU UCUGAUG-3′) were used (Roche Diagnostics, IN).

### Cell culture and transfection

HK-2 cells (human kidney proximal tubular cells) were cultured in Dulbecco’s modified Eagle’s medium/F12 medium (Life Technologies, Carlsbad, CA), which contains 5% FBS (Invitrogen) and 1% antibiotics (100 U/ml penicillin and 100 μg/ml streptomycin) (Life Technologies). The cells were incubated at 37 °C in a humidified incubator with 5% CO_2_. To over-express or down-regulate the expression of specific miRNAs, cells were transiently transfected with miRNA mimics or inhibitor (Life Technologies) at 100 nM concentrations by using the Lipofectamine 3000 (Invitrogen) for indicated time points, according to the manufacturer’s instructions. The negative control contained a scrambled sequence. For TGF-β-treated experiment, cells were cultured in serum-free medium in the presence or absence of 5 ng/mL recombinant human TGF-β1 (R&D Systems, Minneapolis, MN) for different time points.

### Prediction and functional annotation of target gene

Target predictions of common DE miRNAs were conducted using the prediction algorithm Targetscan. To perform annotations of predicted target genes, we utilized the NIH David resource, the Functional Annotation Chart feature with annotations for GO (Gene Ontology) analysis and KEGG (Kyoto Encyclopedia of Genes and Genomes) pathway analysis [25]. The GO covers three domains: Biological Process, Cellular Component, and Molecular Function. Probabilities are evaluated by Bonferroni correction, and false discovery rate (FDR = adjusted *p*-values) values < 0.05 for GO terms and *p* values < 0.01 for KEGG pathway analysis were considered as significant.

### Validation of target genes

Fifty-one putative target genes of hsa-miR-3607-3p and 24 of hsa-miR-4709-3p in the most relevant signaling pathways were validated by qRT-PCR. Total RNA was isolated from HK-2 cells transfected with hsa-miR-3607-3p or hsa-miR-4709-3p mimics, then used to synthesize cDNA using M-MLV Reverse Transcriptase (Life Technologies). QRT-PCR was carried out with SYBR green Permix Kit (Life Technologies) on ABI 7900 system. The housekeeping genes β-actin was used as the internal control. Primers for putative target genes and β-actin were listed in Additional file 9: Table S2. The relative levels of target genes were calculated using 2^−ΔΔCt^ method. Total RNA was extracted from human FFPE specimens as above for validation of ITGB8 and CALM3 in CKD and normal donor kidney samples. The primers used and the procedures of qRT-PCR are the same as above.

### Immunohistochemistry

To detect the protein expression and location of ITGB8 and CALM3 in kidneys, immunohistochemistry was performed in 4-μm FFPE sections of control subjects and CKD patients using a microwave-based antigen retrieval technique as described previously [24]. The primary antibodies used in this study included ITGB8 (SC-25714, Santa Cruz Biotechnology) and CALM3 (NBP2–15669, Novus Biologicals). After immunostaining, sections were counterstained with hematoxylin and representative pictures were captured using Leica Microscopy (Germany) for each group (*n* = 3).

### Dual-luciferase reporter assay

For construction of wild-type luciferase reporter of target genes, the 623 bp 3′ UTR segments of ITGB8 and 630 bp 3′ UTR segments of CALM3 containing recognition sequences for respective miRNAs were sub-cloned into the downstream of the luciferase reporter in pEZX-MT01 vectors (Fulengen, Guangzhou, China). The mutated vectors were constructed by replacing four nucleotides in the binding sites (Fig. 5a). Cells were cultured in 24-well plates to 70–80% confluence. Wild-type or mutant luciferase reporter and miRNA mimics or negative control at 100 nM of final concentration were co-transfected into HK-2 cells. Cells were then harvested 48 h after transfection and luciferase activities were analyzed by Dual Luciferase Reporter Assay (Promega, Madison, WI). Renilla luciferase activity was normalized to firefly luciferase expression for each sample. Data were given as means ± SEM of three independent experiments.

### Western blot

Western blot was conducted in accordance with standard procedures as previously described [24]. Primary antibodies include those against CALM3 (Novus, Littleton, CO) and ITGB8 (Santa Cruz Biotechnology, Dallas, TX). The protein was visualized with Super Signal Western Pico chemiluminescent substrate (Pierce, Rockford, IL), signals were detected by the LiCor/Odyssey infrared image system (LI-COR Biosciences, Lincoln, NE) and quantified by Image J software (National Institutes of Health). The ratio for the protein examined was normalized to β-actin.

### Cell migration assay

The migratory property of cells was evaluated by a wound healing assay performed as previously described [26]. Briefly, HK-2 cells were cultured to confluence in 6-well plates and then a scratch was made using 1-mL pipette tip (Axygen, Union City, CA). The width of cell-free space was measured after 5 ng/mL TGF-β1 treatment for 0, 24, and 48 h by microscope (Zeiss, Germany).

### Cytoskeleton assay

To examine cytoskeleton assembling, cultured HK-2 cells on 10-mm coverslips were fixed in 4% formaldehyde solution for 10 min at room temperature and then permeated in 0.1% Triton X-100 in PBS for 5 min. After washing with PBS, cells were incubated in the staining solution which contains Alexa Fluor-488 phallotoxins in the dilution of 1:50 for 20 min at room temperature. Samples were mounted in the anti-fading mounting medium (Invitrogen) and F-actin distribution was captured using an inverted laser confocal microscope (Zeiss, Germany).

### Statistical analyses

Data from this study were expressed as mean ± SEM. Statistical analyses were performed using one-way ANOVA followed by Newman-Keuls multiple comparison test from GraphPad Prism 5.0 software (San Diego, CA).

## Results

**Fig. 1:**
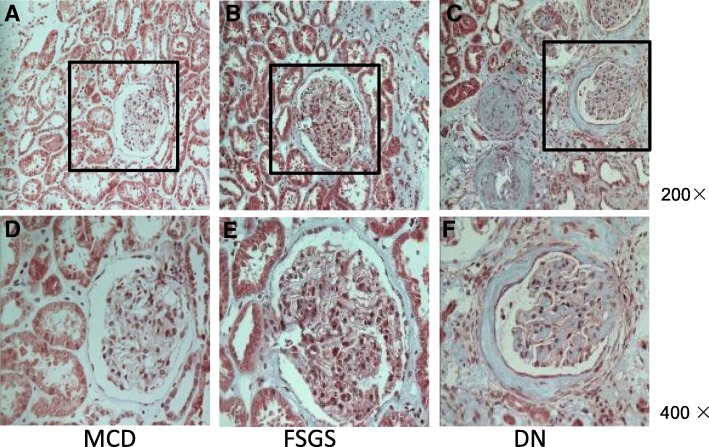
Masson staining of biopsy kidney tissues. MCD, minimal change disease; FSGS, focal segmental glomerulosclerosis; DN, diabetic nephropathy. **a**-**c** are imaged at 200 × magnification; **d**-**f** are imaged at× 400 from the region of black pane in the upper panels

The demographic, clinical, and pathological characteristics of CKD patients are summarized in Table 1, including subjects for microarray screening and PCR validation. The serum creatinine levels for the MCD, FSGS, and DN groups were 65 ± 3.8, 173.4 ± 34.9, 399.6 ± 64.4 μmol/L, respectively. Consistent with the differences in renal function, the pathological changes, including glomerular sclerosis and tubulointerstitial fibrosis, were the mildest in MCD group, moderate in FSGS group, and the most severe in DN group. Representative pictures of Masson’ staining are shown in Fig. 1. There is no significant difference among three groups regarding proteinuria, while serum albumin level was lower in MCD patients compared to FSGS and DN subjects (Table 1).

**Table 1:** Demographic and Clinical characteristics of CKD patients

	MCD	FSGS	DN
Number of cases	10	10	10
Sex (M/F)	6:4	6:4	7:3
Age (year)	26.1 ± 1.5	30.4 ± 2.8	48.6 ± 2.4^a, b^
Serum creatinine (μmol/l)	65 ± 3.8	173.4 ± 34.9	399.6 ± 64.4^a, c^
eGFR (ml/min per 1.73 m^2^)	122.9 ± 4.2	60.3 ± 12.3^a^	20.0 ± 5.3^a, b^
24-h proteinuria (g)	5.3 ± 1.1	3.3 ± 1.0	5.0 ± 1.2
Serum albumin (g/L)	18.1 ± 0.3	36.8 ± 3.8^a^	39.5 ± 1.1^a^
Percentage of glomerular sclerosis (%)	0.8 ± 0.5	41.1 ± 7.3^a^	60.3 ± 7.2^a, d^
Tubulointerstitial fibrosis score	0	2.5 ± 0.2^a^	2.9 ± 0.1^a, d^

*MCD* minimal change disease, *FSGS* focal segmental glomerulosclerosis, *DN* diabetic nephropathy, *M* male, *F* female, *eGFR* estimated glomerular filtration rate. ^a^ versus MCD *p* < 0.001, ^b^ versus FSGS *p* < 0.001, ^c^ versus FSGS *p* < 0.01, ^d^ versus FSGS *p* < 0.05

### MiRNA expression profile in kidney specimens of CKD patients

**Fig. 2:**
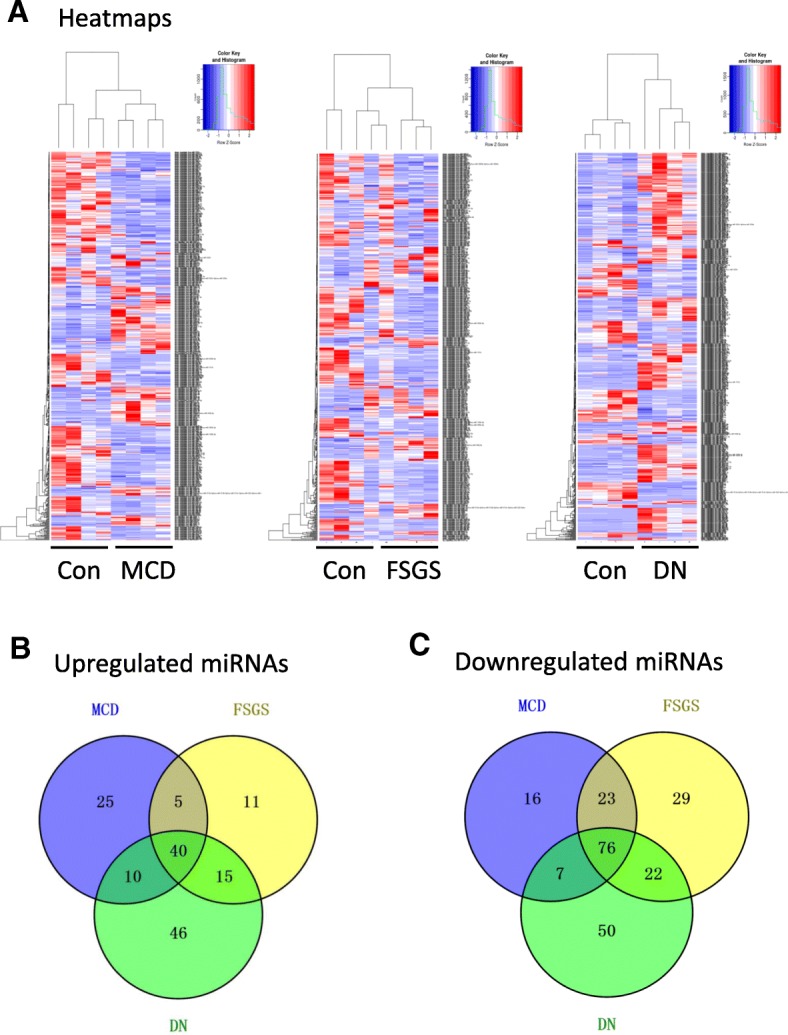
Number of DE miRNAs in kidney biopsy tissues of three CKD groups. **a** The expression of these miRNAs in three CKD groups is illustrated in the heatmap (*n* = 4 in each group). The miRNAs with fold change > 1.5 and *p*-value < 0.05 for expression in CKD patients compared to normal control were considered as differentially expressed (DE). **b** Venn diagram has shown the number of upregulated miRNAs in three CKD groups. Forty miRNAs were elevated in CKD patients compare to normal control. **c** The number of downregulated miRNAs in three CKD groups. Seventy-six miRNAs were decreased in CKD groups compare to normal. MCD: minimal change disease; FSGS: focal segmental glomerulosclerosis; DN: diabetic nephropathy; Con: normal kidney tissues from healthy donor

Total 16 fresh kidney biopsy samples were analyzed for intra-renal miRNA expression profile by microarray screening and the results are presented in Fig. 2 and Additional file 5. A large number of miRNAs were found differentially expressed (DE) in CKD patients compared to control subjects and fold change of these DE miRNAs varied greatly (Fig. 2a). As the Venn diagram (Fig. 2b and c) showed, 80, 71, and 111 miRNAs were up-regulated, while 122, 150, and 155 miRNAs were down-regulated in MCD, FSGS, and DN group, respectively, compared to control group. By comparison of the DE miRNAs profiles among these three CKD groups, 116 miRNAs were found to be commonly dysregulated, with 40 up-regulated and 76 down-regulated as compared with control subjects (Additional file 9: Table S3).

### Validation of DE miRNAs by qRT-PCR in CKD patients and animal model

**Fig. 3:**
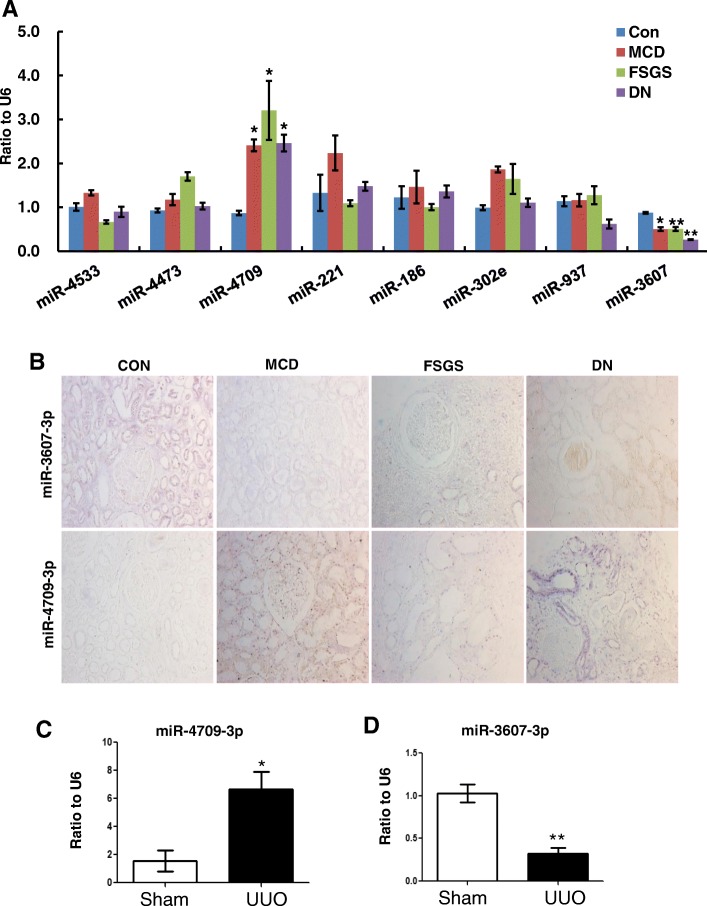
Expression of candidate miRNAs in fibrotic tissues. **a** qRT-PCR validation of top 8 candidate miRNAs in CKD patients. * *p* < 0.05, **p < 0.01 versus control group (*n* = 6 in each group). **b** ISH data using human FFPE sections show that both hsa-miR-4709-3p and has-miR-3607-3p were mainly expressed in tubular epithelial cells. qRT-PCR shows the expression levels of miRNAs in UUO model. Hsa-miR-4709-3p was highly expressed (**c**) but hsa-miR-3607-3p was decreased (**d**) in UUO kidney. MCD: minimal change disease; FSGS: focal segmental glomerulosclerosis; DN: diabetic nephropathy; Con: normal kidney tissues from healthy donor. * *p* < 0.05, **p < 0.01 versus sham group. Data represent means ± SEM for six mice in each group

Top 8 commonly dysregulated miRNAs were selected for validation by qRT-PCR assay using kidney tissues from FFPE sections and the results are shown in Fig. 3a. Two miRNAs, hsa-miR-4709-3p and hsa-miR-3607-3p, were confirmed to changed consistently among all diseased groups. Hsa-miR-4709-3p was found to be up-regulated at 2.5–4 folds, while hsa-miR-3607-3p was down-regulated to 30–50% in CKD samples compared to control subjects. ISH data of these two miRNAs using human FFPE sections showed that both hsa-miR-4709-3p and has-miR-3607-3p were mainly expressed in tubular epithelial cells (Fig. 3b and Additional file 3: Figure S3A). The expression of hsa-miR-4709-3p was markedly up-regulated in the CKD groups, especially in the diabetic kidneys, as compared to control group. On the contrary, has-miR-3607-3p was constitutively expressed in tubular cells of control samples, while largely lost in diseased kidneys. Lastly, we validated the expression of these two miRNAs in mouse UUO model, which is a well-characterized animal model with progressive fibrosis lesions in the kidney. As shown in Fig. 3c and d, miR-4709-3p was significantly increased while miR-3607-3p was largely decreased in the fibrotic kidney of UUO model, which is in agreement with the data from human specimens.

### Annotation analysis of target genes of candidate miRNAs

Hsa-miR-3607-3p and hsa-miR-4709-3p are two newly-found miRNAs which can only be documented in the database of TargetScan. Therefore, in order to characterize their potential biological functions, putative targets genes of these two common miRNAs were predicted by TargetScan and then subjected to GO analysis and KEGG pathway enrichment analysis. Hsa-miR-3607-3p and hsa-miR-4709-3p were predicted to target a total number of 735 and 309 genes respectively (Additional file 6 and Additional file 7). Among 735 predicted targets of hsa-miR-3607-3p, the results of GO classification showed that the number of significant GO_BP (GO terms of biological process), GO_CC (GO terms of cellular component) and GO_MF (GO terms of molecular function) is 168, 25, and 3, respectively (Additional file 6). The most significantly enriched GO_BP terms included regulation of cellular process, cellular process, and biological regulation (Additional file 1: Figure S1A). The top significant KEGG pathways included axon guidance, glutamatergic synapse, ErbB signaling pathway, and regulation of actin cytoskeleton (Additional file 1: Figure S1B). For the 309 target genes of hsa-miR-4709-3p, the number of significant GO_BP terms is 6, whereas none of GO_CC and GO_MF terms were significant (Additional file 7). The top significant GO_BP terms included protein autophosphorylation, generation of neurons, and neuron differentiation (Additional file 2: Figure S2A). The most significantly enriched KEGG pathway was mTOR signaling pathway (Additional file 2: Figure S2B).

### Validation of target genes of hsa-miR-3607-3p and hsa-miR-4709-3p

**Fig. 4:**
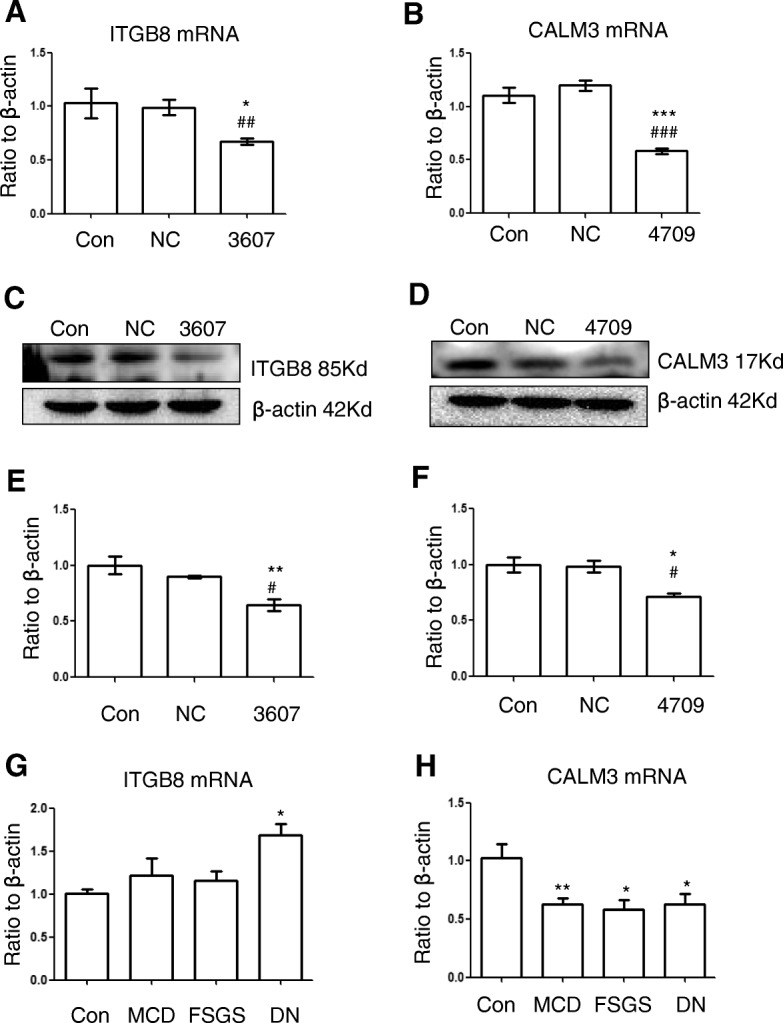
Upregulation of miRNA inhibits the expression level of target genes in HK-2 cells and CKD patients. qRT-PCR shows that transient transfection with hsa-miR-3607-3p (**a**) and hsa-miR-4709-3p (**b**) inhibit the mRNA expression of ITGB8 and CALM3 in HK-2 cells respectively. Western Blot shows that hsa-miR-3607-3p (**c**) and hsa-miR-4709-3p (**d**) inhibit the protein level of ITGB8 and CALM3 respectively. NC: scrambled negative control transfection; 3607: hsa-miR-3607-3p transfection; 4709: hsa-miR-4709-3p transfection. Western Blot shows that hsa-miR-3607-3p and hsa-miR-4709-3p. Quantitative data of relative protein level of ITGB8 (**e**) and CALM3 (**f**) in miRNA mimics transfected cells (normalized to β-actin). Data represent means ± SEM for at least three independent experiments. *p < 0.05, **p < 0.01, ***p < 0.001 versus con; ^#^*p* < 0.05, ^###^*p* < 0.001 versus NC. QRT-PCR of relative mRNA level of ITGB8 (**g**) and CALM3 (**h**) in CKD patients (normalized to β-actin). MCD: minimal change disease; FSGS: focal segmental glomerulosclerosis; DN: diabetic nephropathy; Con: normal kidney tissues from healthy donor. * *p* < 0.05, **p < 0.01 versus control group (*n* = 6 in each group)

**Fig. 5:**
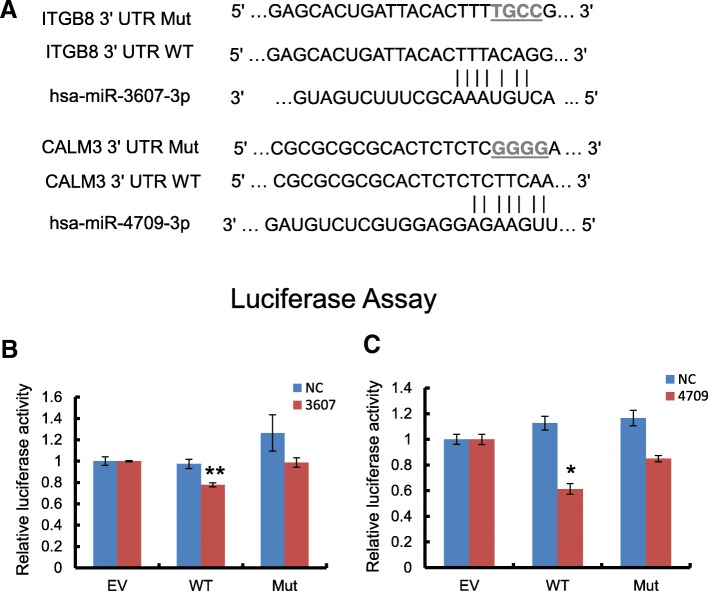
Overexpression of miRNA mimics inhibits luciferase reporter activity in HK-2 cells. **a** The schematic luciferase reporter constructs. A mutant construct was made by replacing four nucleotides in the miRNA seed binding site of target gene 3’UTR. Mutated nucleotides were marked in gray and underlined. The luciferase reporter assay of ITGB8 (**b**) and CALM3 (**c**) 3’UTR reporter in HK-2 cells at 48 h after transfection. EV: empty vector; WT: wild-type; Mut: mutant; NC: scrambled negative control transfection.*p < 0.05 versus NC. Data represent mean ± SEM for at least three independent experiments

Subsequently, we examined the expression of 51 putative target genes of hsa-miR-3607-3p and 24 of hsa-miR-4709-3p in the most relevant signaling pathways (Additional file 8) in hsa-miR-3607-3p or hsa-miR-4709-3p over-expressed HK-2 cells. Results of qRT-PCR and western blot showed that overexpression of hsa-miR-3607-3p or hsa-miR-4709-3p significantly decreased the expression of ITGB8 or CALM3 respectively, at both mRNA and protein levels (Fig. 4a-f). The expression level of ITGB8 or CALM3 was also detected in CKD patients. As shown in Fig. 4g-h, the mRNA level of CALM3 was significantly down-regulated in CKD kidneys compared to control subjects. ITGB8 was mildly increased in MCD and FSGS group, while significantly up-regulated in DN group compared to control. Immunostaining revealed that both ITGB8 and CALM3 were mainly expressed by tubular epithelial cells. The protein level of ITGB8 was up-regulated in diseased kidneys compared to control subjects, while CALM3 showed the opposite trend, both of which coincide with the respective mRNA expression pattern (Additional file 3: Figure S3B). Then we performed luciferase reporter assay to assess the direct regulation of these two miRNAs on their target genes. As shown in Fig. 5, transfection of hsa-miR-3607-3p or hsa-miR-4709-3p mimics into HK-2 cells significantly attenuated the activities of wild-type 3′ UTRs of ITGB8 or CALM3 respectively. However, these inhibitory effects were abrogated when the mutated reporters were applied in which the predicted binding sites for respective miRNAs were mutated. These results demonstrated that ITGB8 and CALM3 were the bona fide target genes of hsa-miR-3607-3p and hsa-miR-4709-3p respectively (Additional file 4).

### Role of hsa-miR-3607-3p and hsa-miR-4709-3p in TGF-β1-induced migration and F-actin assembling of tubular epithelial cells (TECs)

**Fig. 6:**
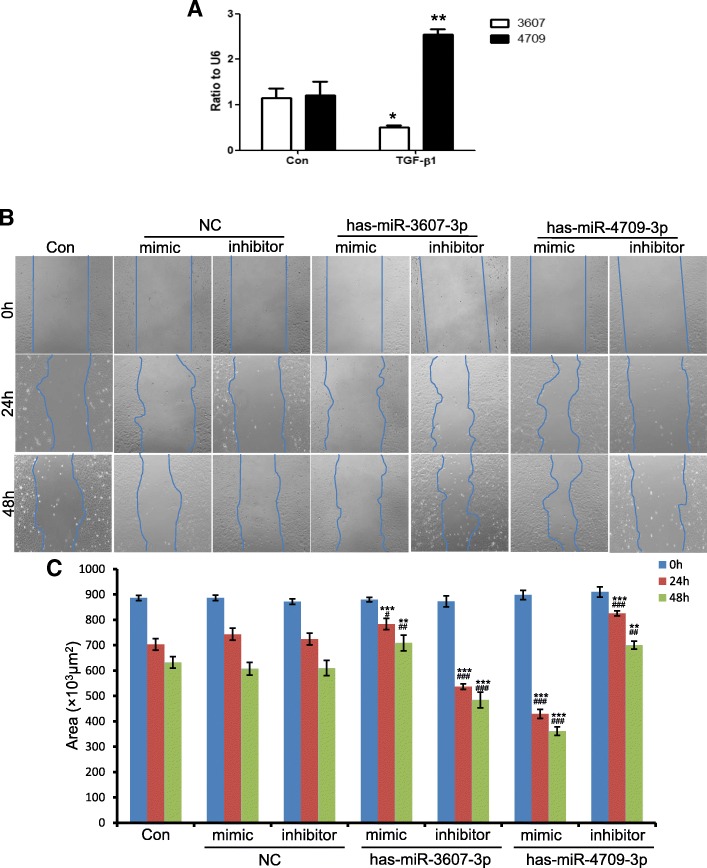
Role of hsa-miR-3607-3p and hsa-miR-4709-3p in TGF-β1-induced migration. **a** Results of qRT-PCR show the expression of hsa-miR-3607-3p and hsa-miR-4709-3p after stimulation by 5 ng/mL TGF-β1 in HK-2 cells. *p < 0.05, *p < 0.01 versus control group. **b** Representative images of the wound healing assay. HK-2 cells were treated with 5 ng/mL TGF-β1 for 0, 24, and 48 h. Cells transfected with negative control (NC), hsa-miR-3607-3p and hsa-miR-4709-3p mimics or inhibitor and then treated by TGF-β1. Original magnification: × 100. **c** Quantitative analysis of the area of cell-free space post-scratch at 0, 24, and 48 h. Data represent mean ± SEM for at least three independent experiments. ***p < 0.01, ***p < 0.001 versus con; ^##^*p* < 0.01, ^###^*p* < 0.001 versus NC at 24 or 48 h

Because both miRNAs were mainly expressed in TECs, this cell type was selected for in vitro mechanistic study. TGF-β1 is the dominant mediator responsible for renal fibrosis and capable of inducing TECs into a fibrotic intermediate cell type, one main characteristic of EMT. Therefore, we firstly examined whether TGF-β1 could modulate the expression of hsa-miR-3607-3p and hsa-miR-4709-3p in vitro. As shown in Fig. 6a and b, TGF-β1 treatment led to a 2.5-fold increase of hsa-miR-4709-3p at 1 h and a 50% decrease of hsa-miR-3607-3p at 24 h, indicating that TGF-β1 regulates their transcription. Next, we examined the effects of these two miRNAs on the cell migratory activity because cells that have higher mobility are in an intermediate state during the fibrosis process. As shown by the wound healing assay in Fig. 6c and d, transfection of hsa-miR-3607-3p mimics or hsa-miR-4709-3p inhibitor into HK-2 cells significantly blocked TGF-β1-induced cell migration. By contrast, inhibition of hsa-miR-3607-3p or overexpression of hsa-miR-4709-3p promoted the cell migratory activities induced by TGF-β1 in HK-2 cells.

**Fig. 7:**
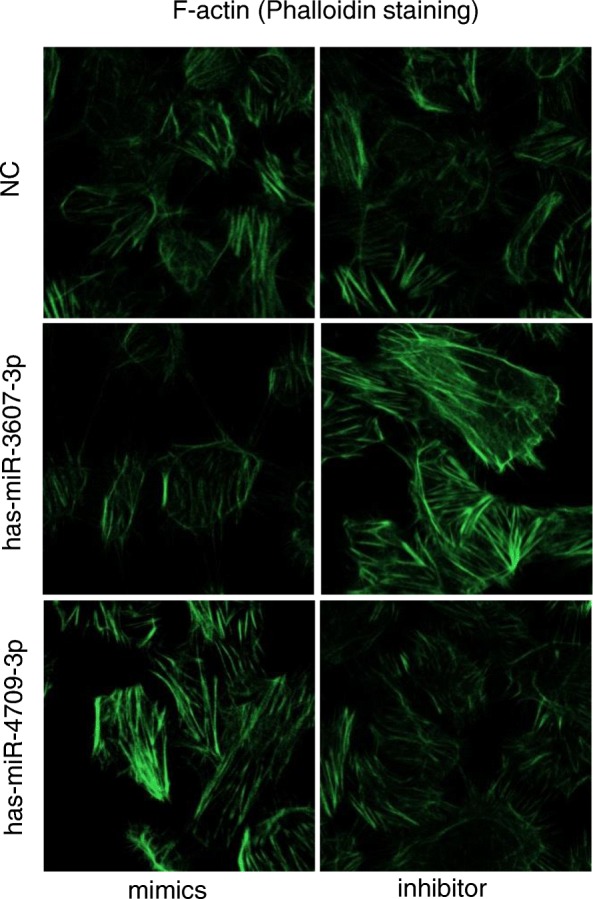
Representative images of F-actin assembling (400×). Phalloidin Alex-488 (green) staining had shown F-actin morphology and distribution in HK-2 cells. Cells were transfected with miRNA mimics or inhibitors and then exposed to 5 ng/mL TGF-β1 for 24 h

In the process of fibrosis, TGF-β is able to rearrange F-actin into parallel stress fiber types which would promote higher cell mobility. As shown by phalloidin staining in Fig. 7, TGF-β1 treatment induced the change of F-actin morphology and assembling in HK-2 cells. Transfection of hsa-miR-3607-3p inhibitor or hsa-miR-4709-3p mimics greatly increased TGF-β1 induced F-actin rearrangement, whereas overexpression of hsa-miR-3607-3p or inhibition of hsa-miR-4709-3p largely blocked stress fiber assembling.

## Discussion

The present study performed microarray profiling of renal biopsy specimens from three different pathological types of CKD patients. We identified that 40 miRNAs were commonly up-regulated while 76 were commonly down-regulated in CKD renal tissues. Two novel miRNAs, hsa-miR-3607-3p and hsa-miR-4709-3p, were verified as consistently differentially expressed in human biopsy samples as well as in UUO fibrosis model. Further functional studies suggested that these two miRNAs might be involved in the CKD-related fibrosis process by rearranging actin cytoskeleton of resident renal cells.

The associations between miRNAs and renal diseases have been extensively investigated [27]. Intra-renal miRNAs expression profiles are found to associate with clinical parameters and histopathological changes in IgAN [10, 11], DN [12, 13], HTN [14], and LN [15]. In this study, we have adopted a novel screening strategy. By comparing three types of renal diseases with different degrees of fibrosis, we identified a unique miRNAs profile that probably related to progression of the common kidney fibrotic process. Some of these DE miRNAs have been widely reported as key regulators in renal fibrosis, including members of miR-21 [13, 17], miR-29 [18, 28], and miR-200 family [20, 29]. Consistent with the well-known anti-fibrotic effect of miR-29, our microarray data showed that hsa-miR-29-5p was down-regulated in both FSGS and DN group compared to control subjects. We also found that hsa-miR-200c-3p was significantly decreased in all three types of CKD, whereas hsa-miR-200a-3p was inhibited in FSGS group, as compared to control group. This is in agreement with the inhibitory role of miR-200 family on EMT in initiation of renal fibrosis. However, two members of miR-21 family, hsa-miR-21-5p and hsa-miR-21-3p, were consistently found to be down-regulated in CKD groups, which contrasts with the known pro-fibrotic role of miR-21 family in the literature [13, 17, 30]. The discrepancies may be due to differences in tissue types, sample processing, isolation and quantification methodology. On the other hand, our study also identified many new miRNAs that have never been described in renal diseases, including hsa-miR-3607-3p and hsa-miR-4709-3p. We selected top 8 common DE miRNAs for validation by qRT-PCR. Among them, hsa-miR-3607-3p and hsa-miR-4709-3p showed the same change trends among all three CKD types consistent with microarray analysis, while other miRNAs were only confirmed in one or two specific disease groups. The similar expression pattern of the above two miRNAs in UUO animal model supported the validity of the microarray screen. The discrepancy of candidate miRNAs expression measured between microarray and qRT-PCR may be due to different sample types we used as well as distinct detection sensitivity for these two techniques.

The functional roles of hsa-miR-3607-3p and hsa-miR-4709-3p in human diseases were poorly characterized. It is reported that miR-3607-3p showed a phased expression pattern at different time points after HIV infection and was implicated in the regulation of cell cycle and T-cell activation [31]. MiR-4709-3p was found to be up-regulated in the serum of chronic HBV-infected patients by deep sequence analysis [32], and might also be specifically involved in hypertension physiology in African American population [33]. The present study added new evidence that these two DE miRNAs might play a role in kidney diseases. As pathway enrichment analysis showed, the significant pathways enriched by predicted target genes of hsa-miR-3607-3p included regulation of actin cytoskeleton, which is a key step in trans-differentiation of resident renal cells and activation of myofibroblasts in the kidney fibrosis process. On the other hand, the most significant enriched pathway for hsa-miR-4709-3p was mTOR signaling pathway, the role of which is well established in diabetes mellitus and its complications, including diabetic nephropathy [34].

In recent years, the role of EMT in renal fibrosis was criticized by several state-of-the-art tracing studies [35, 36] as well as human genomic and transcriptional analysis data [37, 38]. However, we and other investigators believe that to what extent the EMT process contributes to kidney fibrosis is likely to be disease-specific and context-dependent. Using a set of combined indicators within each category of EMT process, a recent study provided strong evidence for an important role of EMT in the development of diabetic nephropathy [39]. EMT involves a change in cell shape, loss of polarity and increased motility associated with increased collagen production [40]. In this study, we also identified that regulation of actin cytoskeleton via targeting ITGB8 and CALM3 may be the mechanisms by which hsa-miR-3607-3p and hsa-miR-4709-3p participate in the kidney fibrosis process. By loss-of- and gain-of-function regulation in vitro, our data confirmed that hsa-miR-3607-3p inhibited whereas hsa-miR-4709-3p promoted actin fibers assembling and cell motility. As demonstrated by qRT-PCR, western blot, and luciferase reporter assay, ITGB8 and CALM3 were found to be the bona fide target genes of hsa-miR-3607-3p and hsa-miR-4709-3p respectively. The ITGB8 gene belongs to the integrin beta chain family and encodes the protein product integrin αvβ8 which is most abundantly expressed in kidney and brain [41]. Integrin αvβ8 is a major receptor for latent TGF-β and is essential for its activation [42]. Our results showed that hsa-miR-3607-3p was decreased in fibrotic kidneys and TGF-β1-treated HK-2 cells, while ITGB8 was inhibited by hsa-miR-3607-3p at both mRNA and protein levels. Therefore, up-regulation of ITGB8 would be expected in renal fibrosis, which might lead to sustained activation of TGF-β signaling, thus forming a positive feedback activation loop. This may be the underlying mechanism by which hsa-miR-3607-3p participate in renal fibrosis. CALM3 encodes the protein product calmodulin-3 which is a core intermediate calcium sensor in calcium signaling pathway [43]. The importance of changes in intracellular calcium levels in cellular morphology changes and actin dynamic during EMT has been documented by a number of studies [44]. In addition, Ca^2+^/calmodulin-dependent protein kinase II, or CaMKII, has also been implicated in EMT in development or cancer metastasis [45, 46]. Therefore, we can speculate that the roles of hsa-miR-4709-3p and CALM3 in kidney fibrosis may be associated with changes in intracellular calcium levels, which may require further research.

There are some limitations in the present study. Firstly, the sample sizes for screening and validation are relatively small so that it is incapable to conduct a convincing correlation analysis between miRNA profiles and clinicopathological data. Those commonly dysregulated miRNAs we identified needs to be verified by other independent studies with larger sample sizes. Secondly, we used microarray rather than next-generation sequencing for screening of miRNA profiles, which prevented the discovery of novel miRNAs or other non-coding RNAs related to renal fibrosis. Thirdly, the heterogeneity among three types of CKD samples makes it difficult to clarify the significance of dysregulated miRNAs. Comprehensive investigations from one single disease at different stages probably produce more intriguing data. Last but not the least, detailed mechanisms by which hsa-miR-3607-3p and hsa-miR-4709-3p participate in specific types of kidney diseases require further exploration.

## Conclusions

In conclusion, the present study identifies commonly dysregulated miRNA profiles related to CKD. Two miRNAs, hsa-miR-3607-3p and hsa-miR-4709-3p, are demonstrated to involve in kidney fibrosis by regulation of actin cytoskeleton rearrangement probably via targeting ITGB8 and CALM3 respectively. Although a limited number of kidney biopsy samples are used in this study, our results may represent a promising research direction for renal disorders and help identify novel biomarkers and therapeutic targets for CKD.

## Additional files

Additional file 1: **Figure S1.** Histogram showing the top ten significant GO terms of biological processes (A) and all the significant KEGG pathways (B) of hsa-miR-3607-3p predicted target genes. (PPTX 73 kb)

Additional file 2: **Figure S2.** Histogram showing the top ten significant GO terms of biological processes (A) and all the significant KEGG pathways (B) of hsa-miR-4709-3p predicted target genes. (PPTX 67 kb)

Additional file 3: **Figure S3.** (A) ISH data using missense probe as negative control of control and CKD sections. (B) Representative images of ITGB8 and CALM3 protein expression as examined by immunohistochemistry. NC: negative control. Original magnification: × 400. (PPTX 4870 kb)

Additional file 4: **Figure S4.** Gene transfer of hsa-miR-3607-3p and hsa-miR-4709-3p in UUO models. (A) Real-time PCR shows that levels of miR-3607-3p and miR-4709-3p are significantly upregulated in the transfection group. (B) H&E (upper panel) and Masson’s trichrome staining (lower panel) of mice kidney. Each bar represents the mean ± SEM for groups of six mice; *P < 0.05, **P < 0.01 versus sham-operated mice; ^#^*P* < 0.05, ^###^*P* < 0.001 versus NC control treatment (UUO + NC). Original magnification: × 400. (PPTX 1110 kb)

Additional file 5: Detailed results of microarray screening profiles using kidney biopsy samples. (XLS 348 kb)

Additional file 6: Results for GO annotation and KEGG pathway enrichment analysis for predicted target genes of miR-3607-3p. (XLSX 215 kb)

Additional file 7: Results for GO annotation and KEGG pathway enrichment analysis for predicted target genes of miR-4709-3p. (XLSX 111 kb)

Additional file 8: Data of validation of putative targets genes of selective miRNAs by qPCR. (XLSX 13 kb)

Additional file 9: **Table S1.** Patient inclusion and exclusion criteria. **Table S2.** Primers used for qRT-PCR validation. **Table S3.** Common dysregulated miRNAs among all CKD biopsy samples. (DOCX 44 kb)

## Data Availability

The data used in the current study is available from the corresponding author on reasonable request.

## Abbreviations

3′ UTRs: 3′ untranslated region
CKD: Chronic kidney disease
DE: Differentially-expressed
DN: Diabetic nephropathy
eGFR: Estimated glomerular filtration rate
EMT: Epithelial-mesenchymal transition
ESRD: End stage renal disease
FFPE: Formalin-fixed paraffin-embedded
FSGS: Focal segmental glomerular sclerosis
GO: Gene ontology
HTN: Hypertensive nephrosclerosis
IgAN: IgA nephropathy
KEGG: Kyoto Encyclopedia of Genes and Genomes
LN: Lupus nephritis
MCD: Minimal change disease
miRNAs: microRNAs
qRT-PCR: quantitative real-time PCR
TECs: Tubular epithelial cells
TGF-β1: Transforming growth factor-β1
UUO: Unilateral ureteral obstruction
