# Supplementary material for: Intrarenal microRNA signature related to the fibrosis process in chronic kidney disease: identification and functional validation of key miRNAs
Source: BMC Nephrol. 2019 Aug 27;20:336. doi: 10.1186/s12882-019-1512-x (PMC6712721; doi:10.1186/s12882-019-1512-x)
Supplement: Supplementary file 1 — Figure S1. Histogram showing the top ten significant GO terms of biological processes (A) and all the significant KEGG pathways (B) of hsa-miR-3607-3p predicted target genes. (PPTX 73 kb) [file 12882_2019_1512_MOESM1_ESM.pptx]

## Slide 1
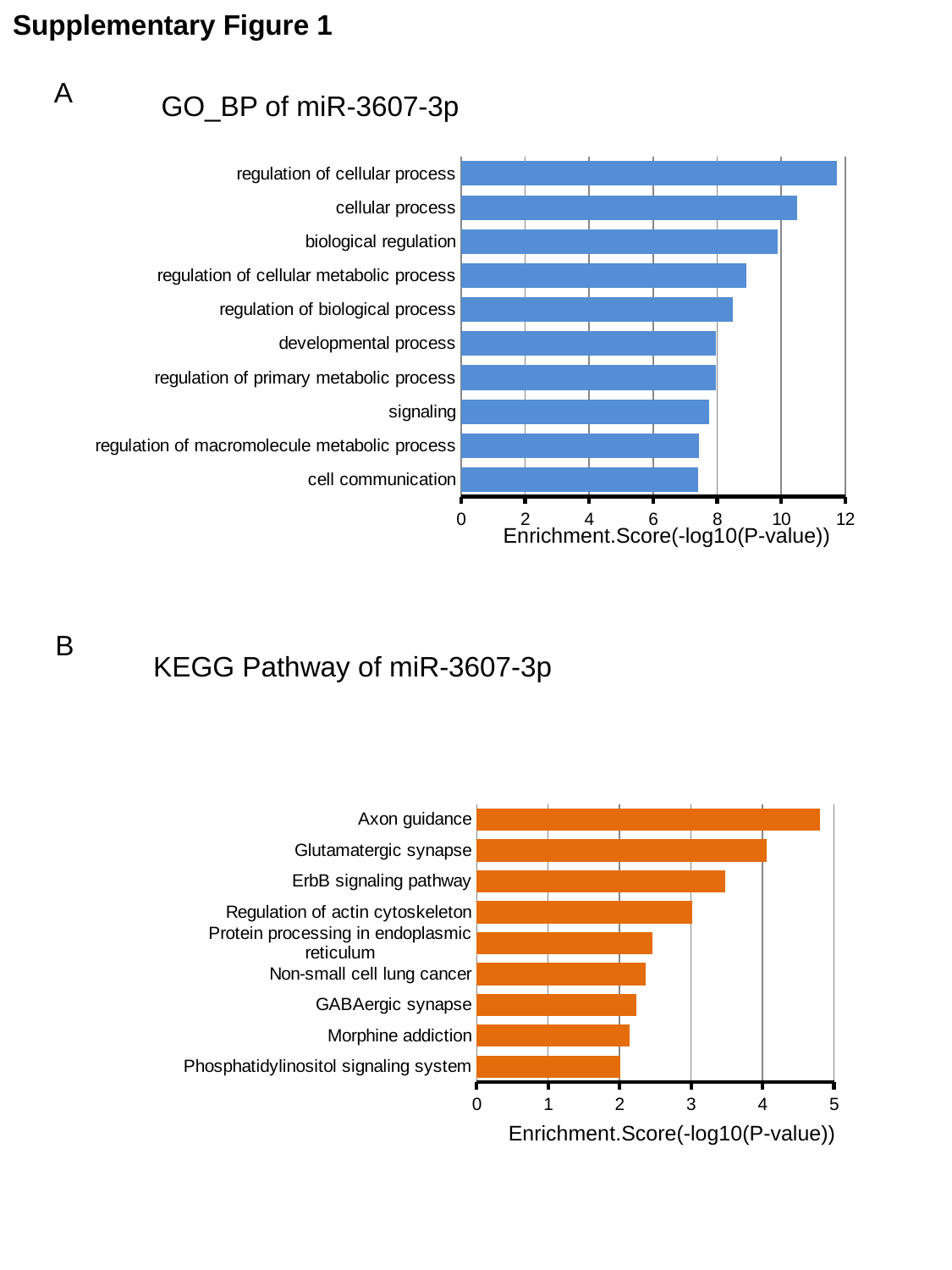

Supplementary Figure 1
A
GO_BP of miR-3607-3p
### Chart
| Category | 3607 |
|---|---|
| cell communication | 7.40946961355349 |
| regulation of macromolecule metabolic process | 7.44523062629729 |
| signaling | 7.7467448833119 |
| regulation of primary metabolic process | 7.96753625803794 |
| developmental process | 7.97335126325363 |
| regulation of biological process | 8.48712135345802 |
| regulation of cellular metabolic process | 8.9055812332485 |
| biological regulation | 9.87839958402692 |
| cellular process | 10.4975639121917 |
| regulation of cellular process | 11.733871408314 |Enrichment.Score(-log10(P-value))
B
KEGG Pathway of miR-3607-3p
### Chart
| Category | 3607 |
|---|---|
| Phosphatidylinositol signaling system | 2.008175 |
| Morphine addiction | 2.14259 |
| GABAergic synapse | 2.23519 |
| Non-small cell lung cancer | 2.36629 |
| Protein processing in endoplasmic reticulum | 2.463567 |
| Regulation of actin cytoskeleton | 3.017019 |
| ErbB signaling pathway | 3.479831 |
| Glutamatergic synapse | 4.061044 |
| Axon guidance | 4.80293 |Enrichment.Score(-log10(P-value))
