# Supplementary material for: Intrarenal microRNA signature related to the fibrosis process in chronic kidney disease: identification and functional validation of key miRNAs
Source: BMC Nephrol. 2019 Aug 27;20:336. doi: 10.1186/s12882-019-1512-x (PMC6712721; doi:10.1186/s12882-019-1512-x)
Supplement: Supplementary file 2 — Figure S2. Histogram showing the top ten significant GO terms of biological processes (A) and all the significant KEGG pathways (B) of hsa-miR-4709-3p predicted target genes. (PPTX 67 kb) [file 12882_2019_1512_MOESM2_ESM.pptx]

## Slide 1
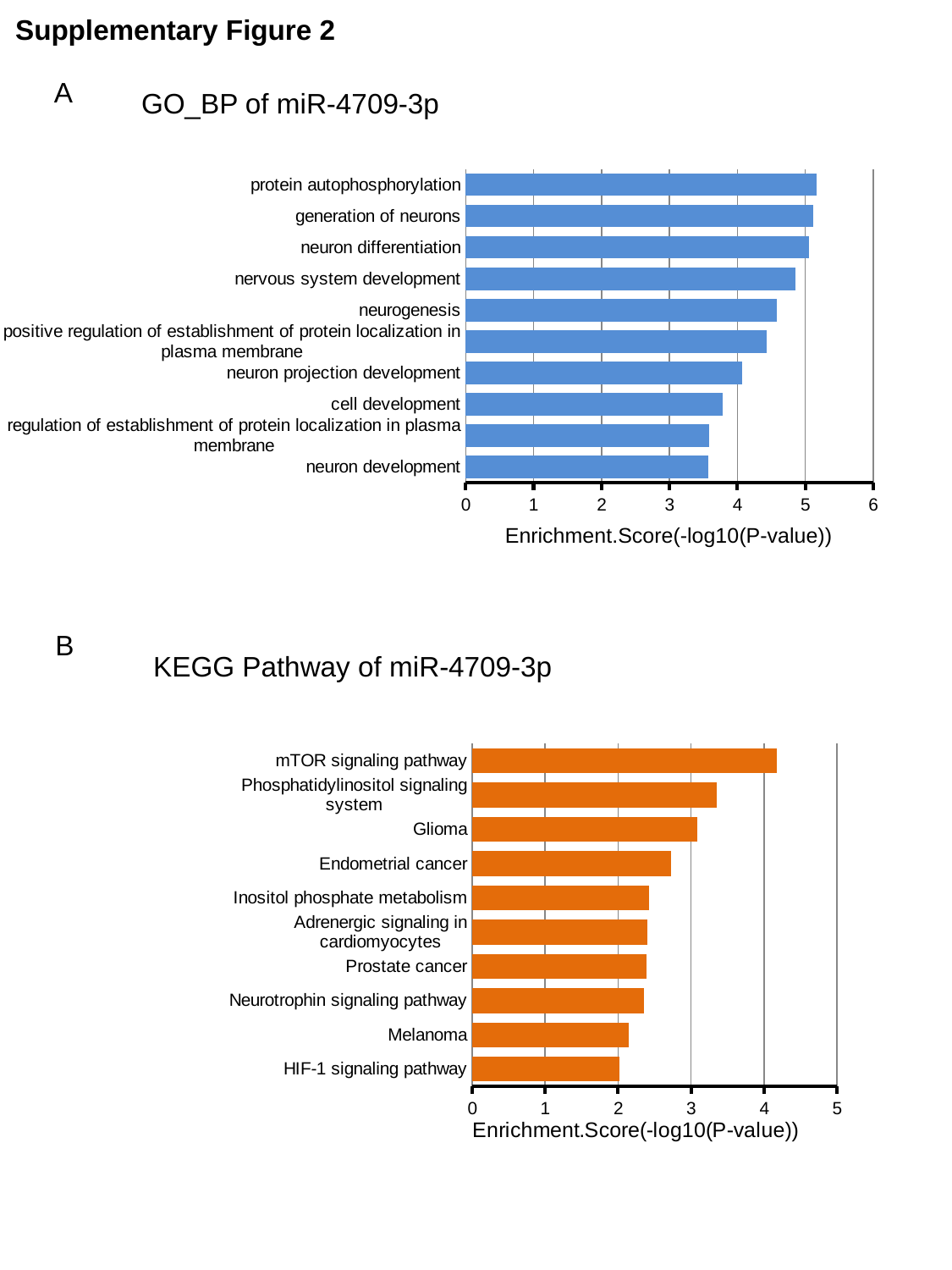

Supplementary Figure 2
A
GO_BP of miR-4709-3p
### Chart
| Category | 4709 |
|---|---|
| neuron development | 3.57043321956628 |
| regulation of establishment of protein localization in plasma membrane | 3.57841924052566 |
| cell development | 3.78519766119593 |
| neuron projection development | 4.0690486931197 |
| positive regulation of establishment of protein localization in plasma membrane | 4.43123319178067 |
| neurogenesis | 4.57556612827205 |
| nervous system development | 4.85389096498147 |
| neuron differentiation | 5.05827527902817 |
| generation of neurons | 5.11620967765179 |
| protein autophosphorylation | 5.17140776621948 |Enrichment.Score(-log10(P-value))
B
KEGG Pathway of miR-4709-3p
### Chart
| Category | 4709 |
|---|---|
| HIF-1 signaling pathway | 2.020386 |
| Melanoma | 2.14041 |
| Neurotrophin signaling pathway | 2.35456299999999 |
| Prostate cancer | 2.38625299999999 |
| Adrenergic signaling in cardiomyocytes | 2.40312 |
| Inositol phosphate metabolism | 2.421786 |
| Endometrial cancer | 2.72836100000001 |
| Glioma | 3.089014 |
| Phosphatidylinositol signaling system | 3.34966599999999 |
| mTOR signaling pathway | 4.175255 |
