# Supplementary figures and images for: Intrarenal microRNA signature related to the fibrosis process in chronic kidney disease: identification and functional validation of key miRNAs
Source: BMC Nephrol. 2019 Aug 27;20:336. doi: 10.1186/s12882-019-1512-x (PMC6712721; doi:10.1186/s12882-019-1512-x)

## Slide 1
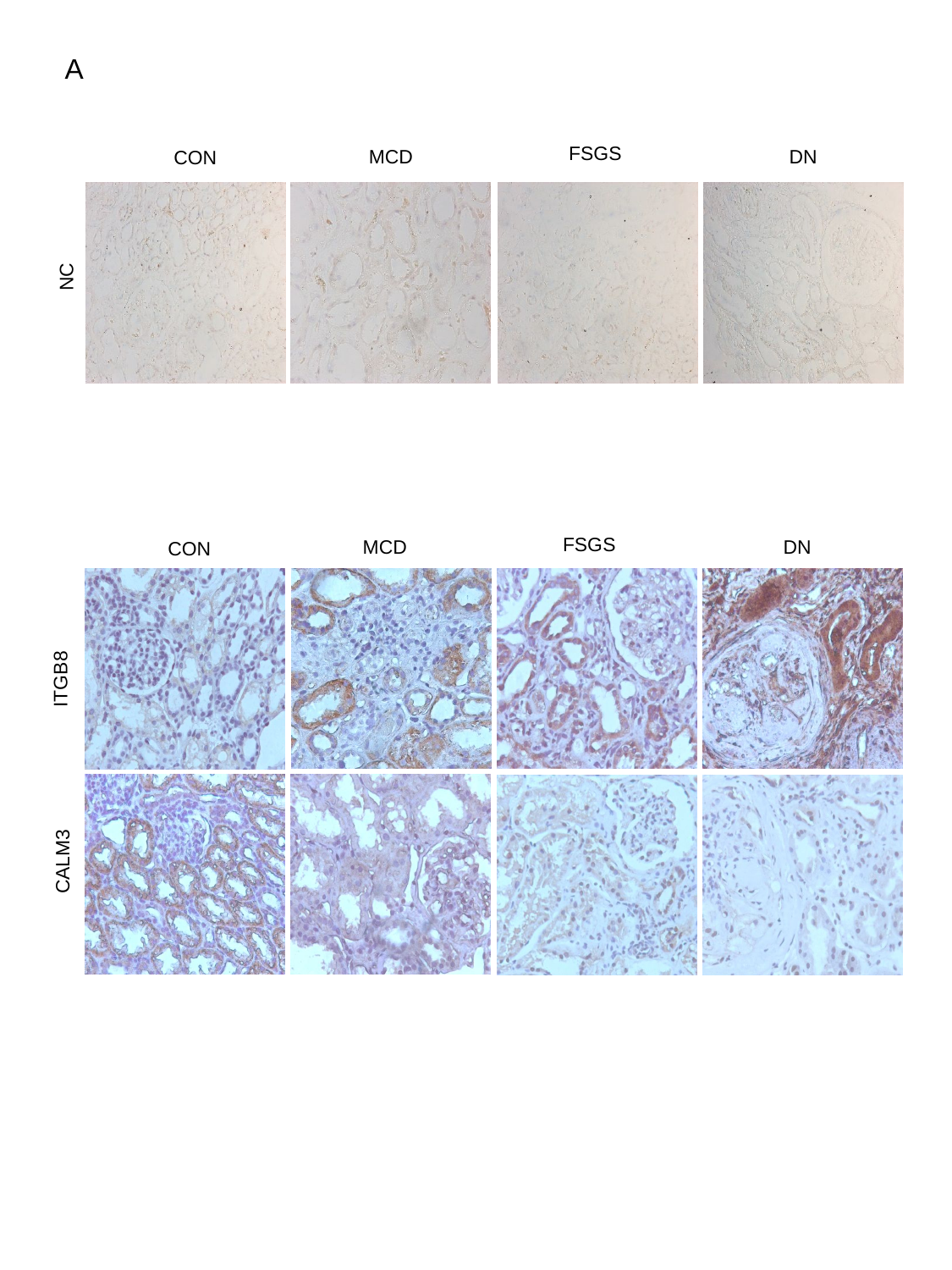

A
FSGS
MCD
DN
CON
NC
FSGS
MCD
DN
CON
ITGB8
CALM3

Supplement: Supplementary file 3 — Figure S3. (A) ISH data using missense probe as negative control of control and CKD sections. (B) Representative images of ITGB8 and CALM3 protein expression as examined by immunohistochemistry. NC: negative control. Original magnification: × 400. (PPTX 4870 kb) [file 12882_2019_1512_MOESM3_ESM.pptx]
