# Supplementary material for: Intrarenal microRNA signature related to the fibrosis process in chronic kidney disease: identification and functional validation of key miRNAs
Source: BMC Nephrol. 2019 Aug 27;20:336. doi: 10.1186/s12882-019-1512-x (PMC6712721; doi:10.1186/s12882-019-1512-x)
Supplement: Supplementary file 4 — Figure S4. Gene transfer of hsa-miR-3607-3p and hsa-miR-4709-3p in UUO models. (A) Real-time PCR shows that levels of miR-3607-3p and miR-4709-3p are significantly upregulated in the transfection group. (B) H&E (upper panel) and Masson’s trichrome staining (lower panel) of mice kidney. Each bar represents the mean ± SEM for groups of six mice; *P < 0.05, **P < 0.01 versus sham-operated mice; #P < 0.05, ###P < 0.001 versus NC control treatment (UUO + NC). Original magnification: × 400. (PPTX 1110 kb) [file 12882_2019_1512_MOESM4_ESM.pptx]

## Slide 1
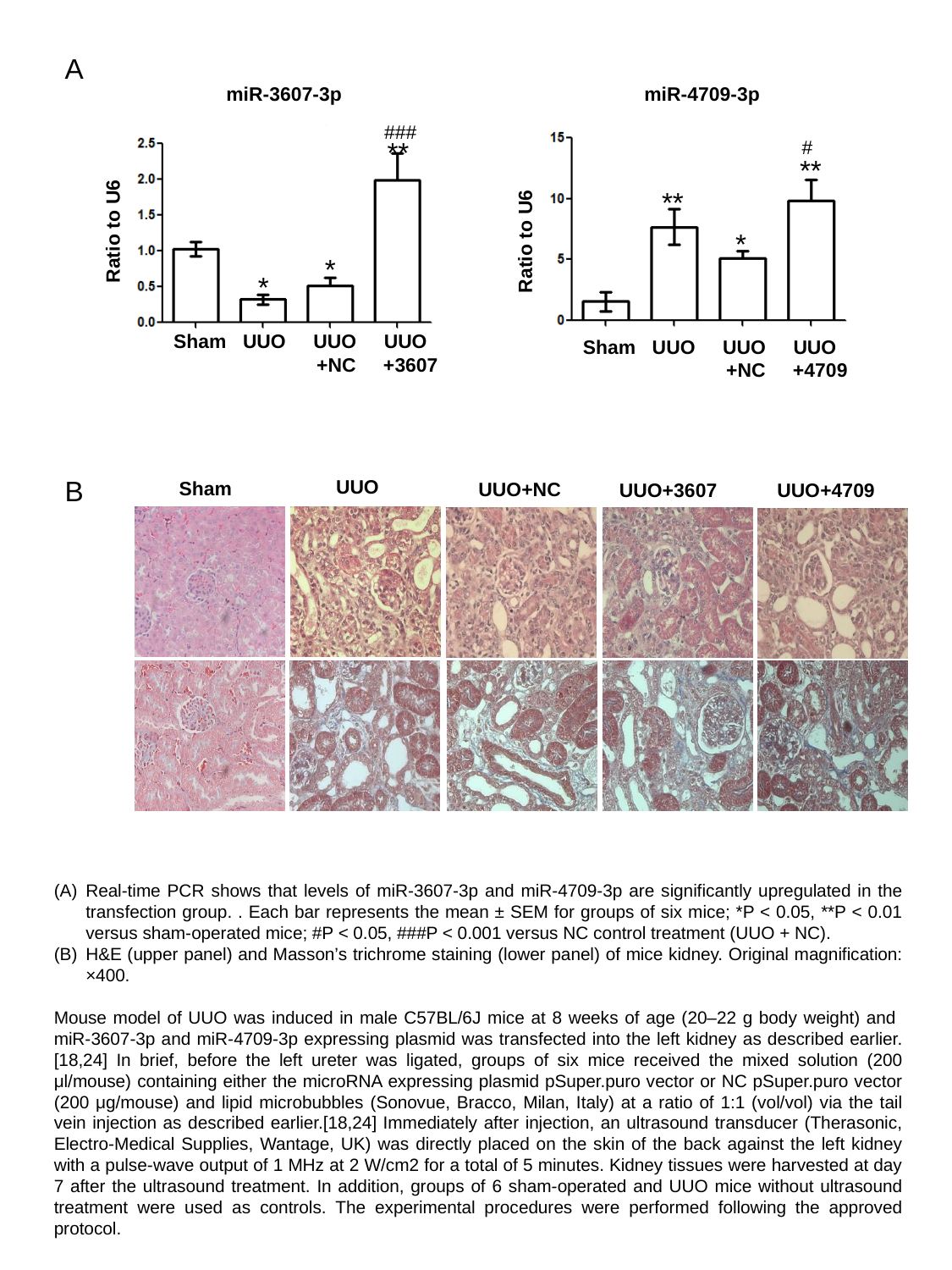

A
miR-4709-3p
miR-3607-3p
###
**
#
**
**
Ratio to U6
Ratio to U6
*
*
*
Sham UUO UUO UUO
 +NC +3607
Sham UUO UUO UUO
 +NC +4709
B
UUO
Sham
UUO+NC
UUO+3607
UUO+4709
Real-time PCR shows that levels of miR-3607-3p and miR-4709-3p are significantly upregulated in the transfection group. . Each bar represents the mean ± SEM for groups of six mice; *P < 0.05, **P < 0.01 versus sham-operated mice; #P < 0.05, ###P < 0.001 versus NC control treatment (UUO + NC).
H&E (upper panel) and Masson’s trichrome staining (lower panel) of mice kidney. Original magnification: ×400.
Mouse model of UUO was induced in male C57BL/6J mice at 8 weeks of age (20–22 g body weight) and miR-3607-3p and miR-4709-3p expressing plasmid was transfected into the left kidney as described earlier.[18,24] In brief, before the left ureter was ligated, groups of six mice received the mixed solution (200 μl/mouse) containing either the microRNA expressing plasmid pSuper.puro vector or NC pSuper.puro vector (200 μg/mouse) and lipid microbubbles (Sonovue, Bracco, Milan, Italy) at a ratio of 1:1 (vol/vol) via the tail vein injection as described earlier.[18,24] Immediately after injection, an ultrasound transducer (Therasonic, Electro-Medical Supplies, Wantage, UK) was directly placed on the skin of the back against the left kidney with a pulse-wave output of 1 MHz at 2 W/cm2 for a total of 5 minutes. Kidney tissues were harvested at day 7 after the ultrasound treatment. In addition, groups of 6 sham-operated and UUO mice without ultrasound treatment were used as controls. The experimental procedures were performed following the approved protocol.
