# Supplementary material for: Intrarenal microRNA signature related to the fibrosis process in chronic kidney disease: identification and functional validation of key miRNAs
Source: BMC Nephrol. 2019 Aug 27;20:336. doi: 10.1186/s12882-019-1512-x (PMC6712721; doi:10.1186/s12882-019-1512-x)
Supplement: Supplementary file 9 — Table S1. Patient inclusion and exclusion criteria. Table S2. Primers used for qRT-PCR validation. Table S3. Common dysregulated miRNAs among all CKD biopsy samples. (DOCX 44 kb) [file 12882_2019_1512_MOESM9_ESM.docx]

**Supplementary Table 1. Patient inclusion and exclusion criteria.**

| **Disease types** | **Inclusion criteria** | **Exclusion criteria** |
| --- | --- | --- |
| MCD | 1.Age between 18 and 70 years; | 1.Secondary MCD(including drug-induced, infection-related MCD, etc.); |
|  | 2.Biopsy-proven MCD; | 2.Family history of nephropathies; |
|  | 3.Renal fibrosis score≤1;^a^ | 3.Usage of immunosuppressive agents prior to biopsy. |
|  | 4.Written informed consent. |  |
| FSGS | 1.Age between 18 and 70 years; | 1.Secondary FSGS; |
|  | 2.Biopsy-proven FSGS; | 2.Family history of nephropathies; |
|  | 3.Renal fibrosis score≥2;^a^ | 3.Usage of immunosuppressive agents prior to biopsy; |
|  | 4.Written informed consent. | 4.Patients with acute kidney injury and/or acute tubulo-interstitial lesions. |
| DN | 1.Age between 18 and 70 years; | 1.Usage of immunosuppressive agents prior to biopsy; |
|  | 2.History of diabetes mellitus and biopsy-proven diabetic nephropathy; | 2.Patients with acute kidney injury and/or acute tubulo-interstitial lesions; |
|  | 3.Renal fibrosis score≥2;^a^ | 3.Patients with co-existing pathological changes(e.g., hypertensive nephropathy). |
|  | 4.Written informed consent. |  |

MCD, minimal change disease; FSGS,focal segmental glomerulosclerosis; DN, diabetic nephropathy.

a: Renal sections were blindly reviewed by an expert pathologist and fibrosis was quantified using the BANFF criteria(1) adapted to the native kidney with a semiquantitative image analysis (0:<10% of cortical area; 1: 10% to 25% of cortical area; 2: 25% to 50% of cortical area; 3: >50% of cortical area)

(1). Racusen LC, Halloran PF, Solez K: Banff 2003 meeting report: New diagnostic insights and standards. Am J Transplant 4: 1562–1566, 2004

**Supplementary Table 2. Primers used for qRT-PCR validation.**

| Gene | Forward | Reverse |
| --- | --- | --- |
| has-miR-4709-3p targets | |  |
| AKT1 | TCCTCCTCAAGAATGATGGCA | GTGCGTTCGATGACAGTGGT |
| CALM3 | GACCATTGACTTCCCGGAGTT | GATGTAGCCATTCCCATCCTTG |
| CREB1 | TTAACCATGACCAATGCAGCA | TGGTATGTTTGTACGTCTCCAGA |
| CAMK2D | GTCACTGAACAACTGATCGAAGC | GAATCGGTGAAAATCCATCCCTT |
| CCND1 | CAATGACCCCGCACGATTTC | CATGGAGGGCGGATTGGAA |
| EIF4B | GGCTGATGAAACGGATGACCT | GGTCGATATTGGGTTCCCGA |
| EIF4E | ATGTGGCGCTGTTGTTAATGT | CTGCGTGGGACTGATAACCAA |
| FGF9 | ATGGCTCCCTTAGGTGAAGTT | CCCAGGTGGTCACTTAACAAAAC |
| IMPAD1 | ACCCACTTGATGCTACACAGG | CACAACGATCCTTGGGGTCT |
| INPPL1 | GCACACGTATCGCATTCTGC | CTCGCTCACCCTCTACAGGAA |
| IRAK3 | CAGCCAGTCTGAGGTTATGTTT | TTGGGAACCAACTTTCTTCACA |
| MAP3K1 | CATCAGGTCGCACAGTGAAAT | TCAGGGCTATATGGTGAGAAGC |
| PFKFB3 | ATTGCGGTTTTCGATGCCAC | GCCACAACTGTAGGGTCGT |
| PIK3R1 | TGGACGGCGAAGTAAAGCATT | AGTGTGACATTGAGGGAGTCG |
| PTEN | TTTGAAGACCATAACCCACCAC | ATTACACCAGTTCGTCCCTTTC |
| PIP4K2B | CCACACGATCAATGAGCTGAG | TCCTTAAACTTAAAGCGGCTGG |
| PLCB3 | TTGAGCGGTTCCTGAACAAG | CACTTCGTTGAGTCTCGGGT |
| PPP2R2C | ACTCCATCTCCGTCAACAGTG | CACCTCCGTAAGGTCCTCCAT |
| SCN5A | TCTCTATGGCAATCCACCCCA | GAGGACATACAAGGCGTTGGT |
| SH2B2 | TGGCTTCCCATCTCAGGAC | CGGCTGGTTCAGAGTAGTGG |
| RRAGC | AGGGCCAATGATGACCTTGC | GTGGAATGAGTTTCTGCACCA |
| ULK2 | GTGGTATTCGCATCAAAATAGCG | CACAAGTCAGCCTTAGCATCATA |
| TCF7 | TTGATGCTAGGTTCTGGTGTACC | CCTTGGACTCTGCTTGTGTC |
| TFRC | GGCTACTTGGGCTATTGTAAAGG | CAGTTTCTCCGACAACTTTCTCT |
| has-miR-3607-3p targets | |  |
| DCC | ACCCAAGCTGGCTTTTGTACT | TGTGACGGCATCAGAAGGTTC |
| DPYSL2 | TGAAGGATCACGGGGTAAATTCC | AATATCCCGGATCACACTCAGT |
| EFNA3 | TCTCTGGGCTACGAGTTCCAC | CCTCAGACACTTCCAGTGCAG |
| EPHA3 | ACTCTACGAGACTGCAATAGCA | TCCCCAAGATCCATTTGAGTGA |
| EPHB2 | GTGTGCAACGTGTTTGAGTCA | ACGCACCGAAAACTTCATCTC |
| GNAI2 | CAGCAAGATGTGCTACGGACC | CACCAAGTCATAGGCGCTCA |
| NFATC3 | GCTCGACTTCAAACTCGTCTT | GATGCACAATCATCTGGCTCA |
| NFATC4 | CTTCTCCGATGCCTCTGACG | CGGGGCTTGGACCATACAG |
| NRP1 | ACGTGGAAGTCTTCGATGGAG | CACCATGTGTTTCGTAGTCAGA |
| PLXNA2 | CTACCTGTCCAGTGTCAACAAG | GGTCGGGAAGTAATCCTGCTT |
| PLXNC1 | CTACAAACTCGTTCCTGATCCTG | GTGGCTGTTAAACACTCCGAA |
| PPP3CA | GCGCATCTTATGAAGGAGGGA | TGACTGGCGCATCAATATCCA |
| SLIT1 | GCCTGGAACTCAATGGCAAC | CTGGTTTCGGTTCAGTCGCA |
| SRGAP1 | ACAAGAAAGACCAGAACCTGTTG | TGCATGAACCGCATAATCACAT |
| UNC5C | ACCTGTACTGTAAAGCAAGCC | GGACAATGAGACCGGAAGTTT |
| UNC5D | CAAGAGCAACCCTATTGCACT | CTCGTTCTGATGGACCCACTC |
| ADCY3 | TTCTCCGAGCCCGAATACTC | GACTCCGGCACGAAAGTCA |
| ADRBK2 | TGAAAGCCTTCGAGGTGACAT | TCGTCCAATAATCCTATGCACAC |
| GLUL | AAGAGTTGCCTGAGTGGAATTTC | AGCTTGTTAGGGTCCTTACGG |
| GNG12 | AGCAAGCACCAACAATATAGCC | AGTAGGACATGAGGTCCGCT |
| GRIA2 | CACCCCACATCGACAATTTGG | GACGTGGAGTGTTCCGCAA |
| GRM3 | AGCAATCACTGGAGTTTGTCAG | GCAATGAGAAGTGGGATGTTTTC |
| HOMER1 | CCGGAAAGTATCAACGGGACA | TCTGAGTTGGTTCAGCCCTTG |
| PLCB3 | repeated |  |
| PRKCB | GGATTGGGATTTGACCAGCAG | TGGCACAGGCACATTGAAGT |
| SHANK3 | AGGACGCGCTCAACTATGG | CTCGCCGCTTGTATCGAAACT |
| SLC38A1 | CACCACAGGGAAGTTCGTAATC | CATCCACGTACCAGGCTGAAA |
| SLC38A2 | ACCGCAGCCGTAGAAGAATG | GCCAGACGGACAATGAGAAGAA |
| ABL2 | TGAAAAGCTACGAGTCCTTGGT | GCTGTTCACTGGGGTGATGTA |
| CBL | TCTACATGAAGTGCATCCCATCA | AAGAGGACCAGGGCTGAAAGA |
| CRKL | CGCTCCGCCTGGTATATGG | GGACACCGACAGCACATAGTC |
| EREG | GTGATTCCATCATGTATCCCAGG | GCCATTCATGTCAGAGCTACACT |
| MAP2K4 | GACGAGGAGCTTATGGTTCTGT | TTTTCATCCACTGTTGACCGAA |
| NRG1 | TGTCACCCAGACTCCTAGCCA | CCTGTGCCATTAAGACGTCCTC |
| NRG2 | CCAGAAGAGGGTCCTGACCAT | GCATCTGCTTCCGCTGTTTT |
| PIK3R1 | repeated |  |
| PIK3R2 | AATGCAGCAAGGAATACCTGG | GCTCTCATGGATCTCGGCAA |
| SOS1 | GAGTGAATCTGCATGTCGGTT | CTCTCATGTTTGGCTCCTACAC |
| EZR | ACCAATCAATGTCCGAGTTACC | GCCGATAGTCTTTACCACCTGA |
| FGF5 | CACTGATAGGAACCCTAGAGGC | CAGATGGAAACCGATGCCC |
| FGF9 | repeated |  |
| GNA12 | GGAGGGATTCTGGCATCAGG | CCGATCCGGTCCAAGTTGTC |
| ITGB3 | AGTAACCTGCGGATTGGCTTC | GTCACCTGGTCAGTTAGCGT |
| ITGB8 | ACCAGGAGAAGTGTCTATCCAG | CCAAGACGAAAGTCACGGGA |
| MYH9 | CCTCAAGGAGCGTTACTACTCA | CTGTAGGCGGTGTCTGTGAT |
| MYL12A | GCTTGCTTCATTGGGGAAGAA | CCTGTATGGTGCCAGTTGCT |
| PFN2 | ATGATTGTAGGAAAAGACCGGGA | GCAGTCACCATCGACGTATAGAC |
| PPP1R12A | GCTGCTAAAGGCTATACGGAAG | AGGTGTCCAGCCATCATAGTC |
| SSH2 | TGGAGCGACACGCTAATTCAT | TGTAAGCTCTGTAGTGCAGACC |
| TIAM1 | GATCCACAGGAACTCCGAAGT | GCTCCCGAAGTCTTCTAGGGT |
| VAV3 | CCAACCCTGGTATGCTGGAG | CCTGTGCCTCACAAGGTAAGT |
| reference gene | |  |
| β-actin | CCCTGAAGTACCCCATCGAGCACG | GGTCATCTTCTCGCGGTTGGCCT |

**Supplementary Table 3. Common dysregulated miRNAs among all CKD biopsy samples.**

|  | MCD |  | FSGS |  | DN |  |
| --- | --- | --- | --- | --- | --- | --- |
|  | FC | p-Value | FC | p-Value | FC | p-Value |
| Up-regulated miRNAs |  |  |  |  |  |  |
| hsa-let-7e-5p | 1.748101 | 0.013683 | 1.868832 | 0.047006 | 2.539058 | 0.000571 |
| hsa-miR-1246 | 5.839751 | 0.00175 | 7.221179 | 0.009602 | 7.21745 | 0.00506 |
| hsa-miR-1299 | 2.362373 | 0.002777 | 2.215484 | 0.036991 | 2.182927 | 0.013077 |
| hsa-miR-205-3p | 9.932827 | 0.000128 | 11.26802 | 0.00027 | 11.21201 | 0.001979 |
| hsa-miR-2116-5p | 4.682766 | 0.000941 | 4.11266 | 0.000626 | 6.832676 | 3.32E-05 |
| hsa-miR-3158-5p | 6.355062 | 0.041737 | 5.598589 | 0.000796 | 14.21816 | 0.011143 |
| hsa-miR-3180-5p | 24.78912 | 0.000405 | 26.74403 | 0.012039 | 33.54251 | 0.017356 |
| hsa-miR-3182 | 4.304137 | 0.002128 | 5.200294 | 0.013644 | 2.703818 | 0.034832 |
| hsa-miR-32-3p | 6.467562 | 0.001343 | 6.147854 | 1.15E-05 | 7.470509 | 0.018484 |
| hsa-miR-335-3p | 2.238499 | 0.00063 | 2.003236 | 0.017441 | 1.920393 | 0.011543 |
| hsa-miR-3686 | 7.942595 | 0.019814 | 8.384666 | 0.001154 | 8.736458 | 0.034383 |
| hsa-miR-371b-5p | 5.484114 | 0.011694 | 7.794949 | 0.008354 | 7.078661 | 0.005638 |
| hsa-miR-3976 | 4.681945 | 0.00069 | 4.845123 | 2.63E-05 | 7.580517 | 5.59E-05 |
| hsa-miR-4308 | 1.809924 | 0.017559 | 1.795897 | 0.030375 | 1.898701 | 0.012286 |
| hsa-miR-4421 | 3.172523 | 0.047383 | 2.390447 | 0.000326 | 3.094019 | 5.27E-05 |
| hsa-miR-4443 | 3.618746 | 0.001458 | 4.222594 | 0.000259 | 5.186199 | 0.002698 |
| hsa-miR-4455 | 3.452361 | 0.048098 | 3.867769 | 0.018705 | 4.543347 | 0.003999 |
| hsa-miR-4456 | 2.091848 | 0.001232 | 2.99318 | 0.003716 | 3.256824 | 0.02342 |
| hsa-miR-4473 | 11.03413 | 0.000672 | 14.56462 | 0.005833 | 13.28991 | 7.34E-08 |
| hsa-miR-4531 | 6.960413 | 0.0002 | 7.514775 | 0.000198 | 11.67996 | 1.68E-06 |
| hsa-miR-4533 | 11.45721 | 0.000686 | 10.45167 | 0.000641 | 19.12945 | 0.001469 |
| hsa-miR-4654 | 3.116139 | 0.03446 | 3.473094 | 0.001725 | 5.031582 | 0.000381 |
| hsa-miR-4682 | 3.265325 | 0.000347 | 3.123259 | 7.85E-05 | 2.420432 | 0.001449 |
| hsa-miR-4685-3p | 8.138458 | 0.014921 | 7.610261 | 0.015334 | 9.014851 | 0.004262 |
| hsa-miR-4708-3p | 3.991924 | 0.014172 | 6.31472 | 0.004791 | 4.541765 | 0.007271 |
| hsa-miR-4709-3p | 10.55375 | 0.002271 | 10.48664 | 0.000449 | 14.13601 | 0.000513 |
| hsa-miR-4726-5p | 4.657248 | 0.005952 | 4.313737 | 0.001246 | 4.754955 | 0.004101 |
| hsa-miR-4747-5p | 6.035521 | 0.009958 | 6.850902 | 0.01227 | 9.269154 | 8.01E-07 |
| hsa-miR-4787-5p | 4.01632 | 0.001777 | 5.174542 | 0.01318 | 4.505184 | 0.005176 |
| hsa-miR-4795-5p | 2.861238 | 0.042545 | 2.403882 | 0.020349 | 2.955636 | 0.002512 |
| hsa-miR-4804-3p | 4.389501 | 0.010041 | 5.170741 | 0.047743 | 3.424144 | 0.010955 |
| hsa-miR-484 | 7.926978 | 0.000626 | 8.86175 | 0.017125 | 13.59945 | 0.024803 |
| hsa-miR-490-3p | 2.614911 | 0.002549 | 2.643873 | 0.001705 | 3.022687 | 0.002397 |
| hsa-miR-5004-3p | 5.212238 | 0.022493 | 4.478094 | 0.006533 | 3.60427 | 0.016046 |
| hsa-miR-5580-3p | 3.003929 | 0.034178 | 2.851164 | 0.024537 | 3.472162 | 0.016784 |
| hsa-miR-664b-3p | 1.790355 | 0.010432 | 1.854744 | 0.000684 | 1.548507 | 0.003906 |
| hsa-miR-758-5p | 5.447132 | 0.011449 | 5.640881 | 0.000896 | 5.776922 | 0.011823 |
| hsa-miR-890 | 3.653416 | 0.01073 | 3.848307 | 0.021822 | 3.229841 | 0.007314 |
| hsv2-miR-H24 | 5.537299 | 0.006325 | 5.889345 | 0.011709 | 8.653055 | 0.028516 |
| kshv-miR-K12-8-5p | 9.396716 | 0.002193 | 10.54408 | 0.022224 | 12.85835 | 0.021398 |
| Down-regulated miRNAs | |  |  |  |  |  |
| ebv-miR-BART7-5p | 0.298383 | 0.029723 | 0.325933 | 0.031014 | 0.259903 | 0.019986 |
| hcmv-miR-UL148D | 0.114313 | 0.009019 | 0.141407 | 0.011196 | 0.327871 | 0.031485 |
| hsa-let-7c-5p | 0.14634 | 0.03046 | 0.194922 | 0.034325 | 0.047389 | 0.035886 |
| hsa-let-7d-3p | 0.101829 | 0.002222 | 0.112254 | 0.002362 | 0.136764 | 0.002744 |
| hsa-miR-10a-5p | 0.103925 | 0.045987 | 0.064881 | 0.038275 | 0.037859 | 0.034579 |
| hsa-miR-1258 | 0.20768 | 0.012729 | 0.088823 | 0.004905 | 0.083187 | 0.001208 |
| hsa-miR-125a-5p | 0.179142 | 0.031263 | 0.164926 | 0.026553 | 0.054233 | 0.01613 |
| hsa-miR-1260a | 0.074219 | 0.018743 | 0.053421 | 0.016948 | 0.043597 | 0.016269 |
| hsa-miR-1260b | 0.38728 | 0.026893 | 0.322271 | 0.011946 | 0.142664 | 0.003213 |
| hsa-miR-1273e | 0.213755 | 0.014657 | 0.296141 | 0.019592 | 0.377596 | 0.048803 |
| hsa-miR-1301-3p | 0.10819 | 0.002023 | 0.204458 | 0.00262 | 0.140692 | 0.002475 |
| hsa-miR-130b-3p | 0.087933 | 3.63E-05 | 0.058432 | 2.51E-06 | 0.309782 | 0.000521 |
| hsa-miR-139-5p | 0.119756 | 0.012664 | 0.112658 | 0.01239 | 0.03138 | 0.008016 |
| hsa-miR-141-3p | 0.200116 | 0.037343 | 0.161823 | 0.028739 | 0.034753 | 0.01614 |
| hsa-miR-145-3p | 0.1668 | 0.02929 | 0.219851 | 0.03759 | 0.147097 | 0.026516 |
| hsa-miR-151a-3p | 0.07794 | 0.008055 | 0.063294 | 0.00714 | 0.066989 | 0.02034 |
| hsa-miR-181a-5p | 0.070544 | 0.040611 | 0.067545 | 0.017312 | 0.022531 | 0.033344 |
| hsa-miR-183-5p | 0.278884 | 0.025979 | 0.155847 | 0.003913 | 0.124733 | 0.000941 |
| hsa-miR-185-5p | 0.112346 | 0.011945 | 0.061545 | 0.000442 | 0.081064 | 0.000492 |
| hsa-miR-186-5p | 0.017634 | 0.022471 | 0.05086 | 0.001973 | 0.028017 | 0.006257 |
| hsa-miR-1976 | 0.081549 | 0.010975 | 0.081968 | 0.010949 | 0.18604 | 0.01875 |
| hsa-miR-200c-3p | 0.234444 | 0.007997 | 0.221691 | 0.006447 | 0.080348 | 0.002763 |
| hsa-miR-204-3p | 0.272194 | 0.00025 | 0.21011 | 9.26E-05 | 0.608975 | 0.00604 |
| hsa-miR-20b-3p | 0.161817 | 0.038304 | 0.199239 | 0.04788 | 0.216954 | 0.04797 |
| hsa-miR-21-5p | 0.078045 | 0.007601 | 0.342143 | 0.030766 | 0.119261 | 0.003109 |
| hsa-miR-221-3p | 0.010852 | 0.021387 | 0.023762 | 0.008391 | 0.01967 | 0.022111 |
| hsa-miR-222-3p | 0.131617 | 0.021279 | 0.144938 | 0.022431 | 0.104405 | 0.018791 |
| hsa-miR-301a-5p | 0.067212 | 0.046963 | 0.072643 | 0.048034 | 0.043264 | 0.042727 |
| hsa-miR-302c-5p | 0.186399 | 0.032525 | 0.074631 | 0.017908 | 0.18028 | 0.032027 |
| hsa-miR-302e | 0.039385 | 0.040119 | 0.026957 | 0.038335 | 0.008307 | 0.035823 |
| hsa-miR-30d-5p | 0.077741 | 0.019148 | 0.084557 | 0.019644 | 0.04971 | 0.016906 |
| hsa-miR-3135a | 0.113014 | 0.012261 | 0.101153 | 0.027706 | 0.162674 | 0.016515 |
| hsa-miR-320a | 0.324339 | 0.004193 | 0.365755 | 0.00177 | 0.320274 | 0.0085 |
| hsa-miR-320b | 0.156265 | 0.002029 | 0.177224 | 0.002158 | 0.165197 | 0.00201 |
| hsa-miR-320c | 0.143473 | 0.003866 | 0.203077 | 0.005035 | 0.110864 | 0.002766 |
| hsa-miR-340-5p | 0.27129 | 0.003361 | 0.308799 | 0.004666 | 0.242033 | 0.00168 |
| hsa-miR-3607-3p | 0.048637 | 0.018477 | 0.039685 | 0.017713 | 0.01229 | 0.015791 |
| hsa-miR-3653 | 0.100259 | 0.006739 | 0.063532 | 0.004996 | 0.040705 | 0.004474 |
| hsa-miR-3664-3p | 0.245029 | 0.001982 | 0.500205 | 0.029118 | 0.472232 | 0.030023 |
| hsa-miR-375 | 0.197122 | 0.005606 | 0.234097 | 0.006501 | 0.237327 | 0.011196 |
| hsa-miR-378d | 0.257955 | 0.036806 | 0.034441 | 0.006248 | 0.005546 | 0.047345 |
| hsa-miR-3915 | 0.435751 | 0.031619 | 0.320244 | 0.010724 | 0.485881 | 0.042657 |
| hsa-miR-423-5p | 0.09858 | 0.003428 | 0.092766 | 0.00335 | 0.172646 | 0.00528 |
| hsa-miR-4255 | 0.303038 | 0.003121 | 0.175154 | 0.001816 | 0.47092 | 0.0347 |
| hsa-miR-4286 | 0.270248 | 0.013161 | 0.263224 | 0.010606 | 0.170462 | 0.006123 |
| hsa-miR-4288 | 0.098547 | 0.010051 | 0.187583 | 0.016228 | 0.093275 | 0.009785 |
| hsa-miR-4300 | 0.14734 | 0.010401 | 0.290992 | 0.040209 | 0.389276 | 0.037356 |
| hsa-miR-4311 | 0.035973 | 0.006308 | 0.003967 | 0.021175 | 0.237555 | 0.010358 |
| hsa-miR-4328 | 0.108999 | 0.014047 | 0.183272 | 0.02013 | 0.086305 | 0.01218 |
| hsa-miR-433-5p | 0.103542 | 0.037475 | 0.056916 | 0.030471 | 0.070121 | 0.032119 |
| hsa-miR-4419b | 0.343726 | 1.71E-05 | 0.391851 | 0.000158 | 0.623635 | 0.00297 |
| hsa-miR-4451 | 0.269896 | 0.023288 | 0.30004 | 0.024116 | 0.395554 | 0.036191 |
| hsa-miR-4497 | 0.170897 | 0.000606 | 0.129732 | 0.000368 | 0.283298 | 0.002257 |
| hsa-miR-4500 | 0.205818 | 0.025402 | 0.254097 | 0.027833 | 0.160921 | 0.019369 |
| hsa-miR-4657 | 0.444791 | 0.003232 | 0.38308 | 0.001088 | 0.317262 | 0.001683 |
| hsa-miR-4714-5p | 0.117398 | 0.017208 | 0.089212 | 0.014894 | 0.078524 | 0.01408 |
| hsa-miR-4716-5p | 0.170572 | 0.016818 | 0.2194 | 0.020145 | 0.363843 | 0.04452 |
| hsa-miR-4728-3p | 0.406439 | 0.019466 | 0.354721 | 0.01148 | 0.473615 | 0.026871 |
| hsa-miR-4775 | 0.304844 | 0.019567 | 0.316394 | 0.01929 | 0.239673 | 0.011054 |
| hsa-miR-500a-5p | 0.249636 | 0.012861 | 0.163788 | 0.00714 | 0.105396 | 0.00487 |
| hsa-miR-505-3p | 0.098641 | 0.028528 | 0.244903 | 0.024348 | 0.103034 | 0.028605 |
| hsa-miR-5571-5p | 0.245964 | 0.024906 | 0.181629 | 0.01751 | 0.181838 | 0.016128 |
| hsa-miR-5701 | 0.240302 | 0.032498 | 0.30632 | 0.045216 | 0.0822 | 0.014335 |
| hsa-miR-574-3p | 0.335598 | 0.045547 | 0.342065 | 0.043513 | 0.300953 | 0.035562 |
| hsa-miR-574-5p | 0.277895 | 0.009352 | 0.3398 | 0.022565 | 0.408673 | 0.035884 |
| hsa-miR-625-3p | 0.067133 | 0.007483 | 0.042897 | 0.001923 | 0.132864 | 0.003216 |
| hsa-miR-634 | 0.556992 | 0.038393 | 0.38683 | 0.004608 | 0.543862 | 0.046752 |
| hsa-miR-647 | 0.255037 | 0.003686 | 0.097075 | 0.004636 | 0.057606 | 0.000615 |
| hsa-miR-7-5p | 0.156817 | 0.000908 | 0.168488 | 0.00075 | 0.45555 | 0.04005 |
| hsa-miR-767-5p | 0.071026 | 0.010082 | 0.050306 | 0.009044 | 0.047787 | 0.009133 |
| hsa-miR-877-3p | 0.234775 | 0.028557 | 0.221671 | 0.026646 | 0.275923 | 0.038274 |
| hsa-miR-891a-5p | 0.395094 | 0.006779 | 0.446662 | 0.016051 | 0.338123 | 0.007356 |
| hsa-miR-92a-3p | 0.063786 | 0.028299 | 0.067495 | 0.027726 | 0.059825 | 0.027022 |
| hsa-miR-937-3p | 0.048189 | 0.015609 | 0.023447 | 0.014027 | 0.010389 | 0.032404 |
| hsa-miR-943 | 0.118842 | 0.000327 | 0.120882 | 0.000236 | 0.25309 | 0.000515 |
| hsv1-miR-H4-3p | 0.086573 | 0.02265 | 0.064502 | 0.020684 | 0.014825 | 0.016626 |

MCD, minimal change disease; FSGS, focal segmental glomerulosclerosis; DN, diabetic nephropathy. FC (Fold Change) cut-off: 1.5; P-value cut-off: 0.05.
